# Current Medical Therapy and Future Trends in the Management of Glaucoma Treatment

**DOI:** 10.1155/2020/6138132

**Published:** 2020-07-21

**Authors:** Barbara Cvenkel, Miriam Kolko

**Affiliations:** ^1^Department of Ophthalmology, University Medical Centre Ljubljana, Ljubljana, Slovenia; ^2^Medical Faculty, University of Ljubljana, Ljubljana, Slovenia; ^3^Department of Drug Design and Pharmacology, University of Copenhagen, Copenhagen, Denmark; ^4^Department of Ophthalmology, Copenhagen University Hospital, Rigshospitalet, Glostrup, Copenhagen, Denmark

## Abstract

Glaucoma is a neurodegenerative disease characterized by progressive loss of retinal ganglion cells and their axons. Lowering of intraocular pressure (IOP) is currently the only proven treatment strategy for glaucoma. However, some patients show progressive loss of visual field and quality of life despite controlled IOP which indicates that other factors are implicated in glaucoma. Therefore, approaches that could prevent or decrease the rate of progression and do not rely on IOP lowering have gained much attention. Effective neuroprotection has been reported in animal models of glaucoma, but till now, no neuroprotective agents have been clinically approved. The present update provides an overview of currently available IOP-lowering medications. Moreover, potential new treatment targets for IOP-lowering and neuroprotective therapy are discussed. Finally, future trends in glaucoma therapy are addressed, including sustained drug delivery systems and progress toward personalized medicine.

## 1. Introduction

Glaucoma encompasses a group of eye conditions, which cause progressive optic nerve damage, retinal ganglion cell death, and corresponding visual field defects. It is the third leading cause of global blindness after uncorrected refractive error and cataract. Glaucoma contributed 8.49% to world blindness among adults aged 50 years and older in 2015 [1]. In the future, the number of glaucoma patients is expected to increase due to growing and ageing populations [2]. More importantly with ageing, time with glaucoma diagnosis will be longer and the lifetime risk of blindness will increase correspondingly. In Sweden, one out of six patients with 12-year median time of diagnosis was bilaterally blind from glaucoma at the last visit [3]. The classification of glaucoma relies on the appearance and obstruction of the drainage pathway and whether it is primary or associated with detectable comorbidity, i.e., secondary glaucoma. The most common type of glaucoma is primary open-angle glaucoma (POAG) with normal, open anterior chamber angle and restricted aqueous outflow associated with increased intraocular pressure (IOP), i.e. high-pressure glaucoma. There is no evidence of a threshold IOP for the onset of glaucoma, but the relative risk for the disease rises with the level of IOP. Nevertheless, most subjects with IOP outside the “normal” range (ocular hypertension) in a population will not develop POAG [4]. In a subtype of POAG, i.e., normal-pressure glaucoma, there is glaucomatous optic neuropathy at the statistically “normal” IOP. It is presumed that risk factors other than IOP have a relatively greater importance and/or sensitivity to IOP may be increased [5, 6]. Even though IOP-lowering therapy delays the onset and progression of glaucoma, the pathogenesis is debatable and not completely understood.

This review aims to (1) summarize current and recently launched IOP-lowering medications, (2) provide a brief overview of new targets for IOP lowering and targets for IOP-independent therapy, and (3) address future trends in therapy.

## 2. Current and Recently Launched IOP-Lowering Medications

IOP is the principle known and modifiable risk factor for development and progression of glaucoma. Hence, reducing IOP has been the mainstay of glaucoma treatment and its lowering by 20–40% has been shown to delay or halt the progression of glaucoma [7–9].

IOP-lowering medications reduce IOP by increasing aqueous outflow and/or reducing aqueous production. There are several types of IOP-lowering eye drops used to treat glaucoma with different mechanisms of action and efficacy (Table 1). The eye drops available in Europe include prostaglandin analogues, *β*-blockers, carbonic anhydrase inhibitors, *α*-2 adrenergic agonists, and parasympathomimetic drugs. In addition, systemic carbonic anhydrase inhibitor drugs are available and can be considered for short time use when eye drops are not effective. Combining agents of different classes with different mechanism of action is associated with superior IOP-lowering efficacy compared to each of the components used alone. Fixed combinations eyedrops in Europe include prostaglandin analogues/*β*-blockers, carbonic anhydrase inhibitors/*β*-blockers, *α*-2 adrenergic agonists/*β*-blockers, carbonic anhydrase inhibitor/*α*-2 adrenergic agonists, and *β*-blockers/parasympathomimetics.

New medications have been approved by the Food and Drug Administration (FDA) in 2017 and not yet by the European Medicines Agency (EMA). These include latanoprostene bunod and netarsudil, and in 2019, fixed combination netarsudil/latanoprost was launched.

### 2.1. Prostaglandin Analogues

Prostaglandin analogues (PGAs) are recommended as first choice treatment for POAG, because of their efficacy, limited systemic side effects, and once daily dosing [10, 11]. Differences among drugs within this class in the IOP reduction did not exceed 1 mmHg [12]. They lower IOP by increasing uveoscleral outflow. The most common side effects are conjunctival hyperaemia, increased pigmentation of periocular skin, longer and thicker eyelashes, and change in iris colour in some eyes (mostly in green-brown or grey-brown eyes) [13, 14]. A few cases of recurrence of herpetic keratitis have been reported with the use of prostaglandins [15].

### 2.2. *β*-Blockers (Adrenergic Antagonists)

These drugs decrease aqueous humour production by blocking *β*-adrenergic receptors in the ciliary epithelium. *β*-Blockers are less effective during night time, because of naturally reduced aqueous humour production at night [16]. Nonselective *β*1 and *β*2 receptor antagonists may have higher efficacy (Table 1) compared to the *β*1-selective antagonist, betaxolol. Ocular adverse effects include conjunctival hyperaemia, epithelial keratopathy, and slight decrease in corneal sensitivity. Systemic adverse reactions include decreased heart rate and cardiac contractility, bronchospasm, depression, impotence, and anxiety [17]. *β*-Blockers should not be used in patients with bradycardia, heart block, manifest cardiac failure, and asthma. Respiratory adverse reactions are mediated via *β*2 receptor blockage. Hence, betaxolol can be considered in cases with respiratory issues [18].

### 2.3. Carbonic Anhydrase Inhibitors (CAIs)

Carbonic anhydrase inhibitors reduce production of aqueous humour by inhibiting carbonic anhydrase in the ciliary epithelium [19]. Systemic CAIs effectively lower IOP, but the adverse effects limit their use for long-term therapy. Common adverse effects include paraesthesia, nausea, vomiting, depression, kidney stones, and metabolic acidosis. Topical CAIs are systemically safe; ocular adverse effects include stinging, burning, foreign body sensation, and, with brinzolamide, transient blurring of vision. Carbonic anhydrase is naturally present in the endothelial cells, and topical CAIs were reported to cause irreversible corneal decompensation in patients with corneal endothelial disorders [20].

### 2.4. Adrenergic Agonists

Adrenergic agonists decrease aqueous humour production and increase uveoscleral outflow. Nonselective adrenergic agonists have been in clinical practice replaced by *α*-2 selective agents, of which brimonidine only is used for chronic therapy. Apraclonidine has been associated with a high rate of allergic blepharoconjunctivitis and is used only for short-term prophylaxis to prevent IOP increase after laser procedures. Ocular adverse effects include allergic reactions and periocular contact dermatitis occurring in 12–15% of patients [21, 22]. Systemic side effects include dry mouth, fatigue, and headache [21]. These agents should not be used in small children, because they cross the blood-brain barrier and can cause respiratory and central nervous system depression. A randomized controlled trial comparing brimonidine versus *β*-blocker timolol in patients with low-tension glaucoma found that patients treated with brimonidine were less likely to have visual field progression than patients treated with timolol [23]. This non-IOP-dependent mechanism suggests a potential neuroprotective role of *α*-2 agonists.

### 2.5. Parasympathomimetics (Cholinergic Drugs)

Parasympathomimetics increase aqueous outflow through trabecular meshwork. Pilocarpine is a direct agonist of parasympathetic receptors, whereas echothiophate is an indirect acting agonist and inhibits acetylcholinesterase. The ocular side effects include miosis, pseudomyopia, brow ache, red eyes, miosis-induced visual field constriction, and decreased vision at night. Miotics may cause increased salivation, sweating, diarrhoea, vomiting, and tachycardia [19].

## 3. Novel IOP-Lowering Medications for Treatment of Glaucoma

### 3.1. Rho-Kinase Inhibitor Netarsudil

Netarsudil is the only available rho-kinase inhibitor, which represents the first new class glaucoma drug in more than 20 years. It has received approval by the FDA in 2017 (Rhopressa™, Aerie Pharmaceuticals, Inc., USA), and recently the European Medicines Agency's Committee for Medicinal Product for Human Use approved the use of netarsudil 0.02% (Rhokiinsa™, Aerie Pharmaceuticals Ireland Ltd.) for treatment of open-angle glaucoma and ocular hypertension [24]. Rho-kinase is a serine/threonine protein kinase that regulates cytoskeletal activities and calcium-dependent smooth muscle contraction. Its functions include modulation of cell adhesion, increasing cell stiffness and contraction of actomyosin, and influencing aqueous humour outflow [25]. Netarsudil lowers resistance to outflow through trabecular meshwork, decreases aqueous production, and decreases episcleral venous pressure. It is supplied as a buffered aqueous solution with a pH∼5 and dosed once daily. In a 28-day clinical trial comparing IOP-lowering efficacy of netarsudil 0.02% versus latanoprost, netarsudil was found to be less effective in patients with open-angle glaucoma and ocular hypertension by approximately 1 mmHg [26]. Other clinical trials compared IOP-lowering efficacy of netarsudil versus timolol, with two studies of three months' and one trial of 12 months' duration [27–29]. Netarsudil was found to be effective, consistently lowering IOP through 12 months, and noninferior to timolol, but with higher incidence of conjunctival hyperaemia and subconjunctival haemorrhages versus both latanoprost and timolol. The ocular adverse effects included conjunctival hyperaemia noted in 48–60%, small microhaemorrhages in or around the limbus in 6–20%, and cornea verticillata in 5–24% of patients. Other ocular side effects include blurred vision, eyelid erythema, instillation-site pain, increased lacrimation, and reduced visual acuity. Conjunctival hyperaemia is due to rho-kinase inhibition of calcium sensitization and leads to blood vessel smooth muscle relaxation and consequently to vessel dilation [27]. The conjunctival hyperaemia is usually mild and was a reason for discontinuation of treatment in 4% of patients [29]. Subconjunctival haemorrhages resolved with continued use of netarsudil. Cornea verticillata was reported to be mild, without impact on vision, and resolved within few months after stopping therapy. Drugs that are both cationic and amphiphilic can induce cornea verticillate, which is due to lysosomal accumulation of phospholipids within corneal epithelial cells, a process called phospholipidosis. Netarsudil is a cationic amphiphilic drug and can induce phospholipidosis [28]. Systemic side effects were not observed with netarsudil.

### 3.2. Nitric Oxide-Donating Prostaglandin Analogue: Latanoprostene Bunod

Latanoprostene bunod is a novel nitric oxide-donating prostaglandin F2*α* receptor agonist that is metabolised to latanoprost acid and butanediol mononitrate, which releases the second active component, nitric oxide. Latanoprostene bunod ophthalmic solution 0.024% (Vyzulta, Bausch & Lomb Incorporated, Rochester, New York, USA) was approved by the FDA in 2017 for the reduction of IOP in patients with open-angle glaucoma or ocular hypertension and is not available in Europe. It has dual mechanism of IOP lowering: latanoprost acid increases uveoscleral aqueous humour outflow and nitric oxide increases trabecular meshwork and Schlemm's canal outflow. Nitric oxide activates the nitric oxide-guanylate cyclase-1-cGMP cascade, resulting in trabecular meshwork relaxation and consequently increased aqueous humour outflow [30]. Nitric oxide is a regulator of blood flow through relaxation of the vascular smooth muscle and has been shown to have either neuroprotective or neurodegenerative effect on retinal ganglion cells in animal models [30–32]. Very high concentrations of nitric oxide caused oxidative damage to the retinal ganglion cells in some animal models [33]. Because of very short half-life of nitric oxide (less than three seconds in extravascular tissues), it is highly unlikely that nitric oxide released from latanoprostene bunod would reach the retina at toxic levels [34]. In two clinical trials with a three months' duration comparing latanoprostene bunod once daily versus timolol twice daily at three time points, latanoprostene bunod achieved significantly lower IOP at all time points [35, 36]. In the pooled analysis of both studies, the percentage of subjects with mean IOP ≤18 mmHg and the percentage with IOP reduction ≥25% were significantly higher in the latanoprostene bunod group versus the timolol group (mean IOP ≤ 18 mmHg: 20.2% vs. 11.2%, *P*=0.001; IOP reduction ≥ 25%: 32.9% vs. 19.0%, *P* < 0.001). Both trials extended as open-label studies showed that patients treated with latanoprostene bunod maintained consistently lowered IOP at 6 and 12 months. Patients switched from timolol to latanoprostene bunod had an additional and sustained decrease in mean diurnal IOP [37]. The adverse effects of latanoprostene bunod are similar to those of prostaglandin analogues and were more frequent than in the timolol group [38]. The most common ocular adverse effects through 1 year of treatment were conjunctival hyperaemia (17.7%), growth of eyelashes (16.2%), eye irritation (11.5%), eye pain (10.0%), and increase in iris pigmentation (9%) [39]. There were no systemic adverse effects related to this drug.

### 3.3. Fixed Combination: Rho-Kinase Inhibitor/Latanoprost

Fixed combination netarsudil 0.02%/latanoprost 0.005% ophthalmic solution (Rocklatan™, Aerie Pharmaceuticals, Inc., USA) is the first fixed combination of a prostaglandin analogue and the rho-kinase inhibitor. It received FDA approval for the treatment of open-angle glaucoma and ocular hypertension in 2019 [40]. Netarsudil lowers IOP by increasing aqueous outflow through trabecular meshwork, decreasing aqueous production, and decreasing episcleral venous pressure, and its mechanism of action complements that of latanoprost which lowers IOP by increasing uveoscleral outflow. Fixed combination is prepared as a buffered aqueous solution with a pH∼5 and dosed once daily. Two clinical trials of 3 months' duration compared fixed combination of netarsudil/latanoprost versus monotherapy with netarsudil or latanoprost [41, 42]. Both studies found that fixed combination showed statistically and clinically significant superior IOP-lowering efficacy compared to its individual components. Fixed combination netarsudil/latanoprost lowered IOP by an additional 1.8–3.3 mmHg compared with netarsudil and 1.3–2.5 mmHg compared with latanoprost. From both clinical trials, mean diurnal IOP reduction of ≥30% was achieved in 58.8–64.5% of patients treated with fixed combination netarsudil/latanoprost, 20.6–28.8% of netarsudil, and 29.4–37.2% of latanoprost groups. Ocular side effects include those related to individual component. Currently there is one ongoing clinical trial in Europe comparing fixed combination netarsudil/latanoprost to fixed combination bimatoprost/timolol (NCT03284853). The most frequent ocular side effects were mild conjunctival hyperaemia (44%), conjunctival haemorrhage (10%), and cornea verticillate (5–13%). Conjunctival hyperaemia was a reason to discontinue therapy in 7% of patients [41, 42]. Other ocular side effects include pain at the site of instillation, increased lacrimation, eye pruritus, and asymptomatic corneal changes. Corneal changes refer to changes in the appearance of endothelial cells with specular microscopy that were found in 5.7% of patients treated with fixed combination netarsudil/latanoprost, 4.7% with netarsudil, and in none with latanoprost [42]. No systemic adverse effects were reported.

The novel drugs netarsudil, a rho-kinase inhibitor, latanoprostene bunod, and fixed combination of netarsudil/latanoprost represent an extension of treatment options with different mechanisms of action. Interestingly, in clinical trial, the new compounds were compared to timolol and not to prostaglandin analogues (except for netarsudil that showed lower IOP lowering compared to latanoprost in a 28-day trial; NCT01731002), and the new fixed combination was compared to its separate individual components and not concomitant treatment. All three drugs are preserved with benzalkonium chloride (BAK). Netarsudil contains 0.015% BAK, and latanoprostene bunod and fixed combination netarsudil/latanoprost contain 0.02% BAK. Because of the well-known toxic-inflammatory effects of BAK, these drugs are not suitable for patients with signs or symptoms of dry eye. Another issue is the cost of medication, which if paid out of the pocket would be an important obstacle for long-term treatment.

## 4. New Targets for IOP-Lowering and for IOP-Independent Therapy

### 4.1. IOP-Lowering Treatment Strategies

Currently, lowering of IOP is the only clinically proven strategy for successful neuroprotection. Depending on the severity of glaucoma at diagnosis and years with the diagnosis, IOP lowering to the individual target delays progression of disease and preserves adequate visual function in most, but not all glaucoma patients. Reduction of IOP removes stress causing glaucomatous optic nerve damage, but it does not stimulate cell survival or cell resilience to withstand pathological insults or prevent cells' death. Ideally, the new targets for glaucoma treatment should include both IOP-lowering and non-IOP-related effects (neuroprotective). Some of the new targets have shown to achieve either one or both effects in animal models of glaucoma, but translation of preclinical results into clinical glaucoma practice is challenging and has so far not been successful.

#### 4.1.1. Cannabinoids

Cannabinoids have been investigated for their IOP-lowering effect for the past few decades. The exact mechanism is incompletely understood. They lower IOP by inhibiting calcium influx through presynaptic channels and in this way reduce the noradrenaline release in the ciliary body, leading to a decrease in the production of aqueous humour [43]. The main active component is ∆^9^-tetrahydrocannabinol (THC) which acts with G-protein-coupled type-1 and type-2 cannabinoid (CB1 and CB2) receptors that are the most important endogenous cannabinoid-binding targets within the so-called “endocannabinoid system” [44]. CB1 and CB2 receptors are present in the central nervous system and within the eye in the retina. CB1 receptors in the trabecular meshwork, Schlemm's canal, and ciliary body are involved in the regulation of IOP [45]. THC's action on CB1 receptors in ciliary body leads to vasodilation of blood vessels in the anterior uvea, favouring aqueous humour efflux [46]. In addition, THC increased retinal ganglion cell survival in an animal model of glaucoma and inhibited glutamate release by increasing K^+^ and decreasing Ca^2+^ permeability [47, 48]. Oral administration of cannabinoids is not a suitable treatment modality, because of side effects, variable absorption, and poor predictability of timing and peak effect. Topical administration could potentially be ideal for glaucoma patients, but at present there is no solid evidence supporting use of cannabinoids for glaucoma [49]. Recently, a hydrophilic prodrug of THC, ∆^9^-tetrahydrocannabinol-valine-hemisuccinate, has been synthesized with the aim to improve the ocular bioavailability of THC [50]. The prodrug formulated in a lipid-based nanoparticle carrier was evaluated in an animal model. It lowered IOP by 30% from baseline at peak and the IOP decrease lasted for six hours.

#### 4.1.2. Melatonin

Melatonin is synthesized by several ocular structures. Melatonin and its analogues decrease IOP by activation of membrane receptors MT1 and MT2, located in ocular tissues, including ciliary processes. Melatonin receptors belong to the G-protein-coupled receptors [51]. In healthy, normotensive eyes, melatonin receptors form complexes with *α*1-adrenoceptors. These functional units couple to Gs which leads to an increase in cAMP levels and protein kinase A activity. In the hypertensive eyes, these functional adrenergic/melatonin receptor units are not formed. The individually expressed *α*1-adrenoceptors allow adrenergic agonists to increase cytosolic Ca^2+^ levels and the expression of individual melatonin receptors, which couple to Gi leading to decrease in cAMP levels and protein kinase A activity. Several analogues were studied for their IOP-lowering effect, which depended on the status of the eye (normotensive or hypertensive) [52, 53]. The most promising melatonin analogue is agomelatine which is used to treat depressive disorders. In glaucoma patients on topical IOP-lowering medication, agomelatine further lowered IOP by 30% [54]. Melatonin has also antioxidant function acting as effective free radical scavenger, and its analogues may have promising application in glaucoma therapy [55].

#### 4.1.3. Connective Tissue Growth Factor

Connective Tissue Growth Factor (CTGF) is a downstream molecule in the Transforming Growth Factor (TGF) *β*-2 signalling cascade. CTGF is a matricellular protein which is expressed by the cells of trabecular meshwork, ciliary body, and retina [56]. Increased levels of CTGF were found in the aqueous humour of patients with the secondary glaucoma subtype, pseudoexfoliation glaucoma [57]. CTGF increases the expression of fibrotic extracellular matrix, fibronectin, and cells' stiffness [58]. Consequently, the trabecular meshwork outflow facility decreases. In a transgenic mice model with overexpression of CTGF, IOP was higher in mice overexpressing CTGF compared to control mice [59]. Inhibiting CTGF-induced extracellular matrix production does not interfere with TGF*β*-2 pleiotropic effects, therefore targeting CTGF may prove beneficial and safer in the treatment of glaucoma. Recently, the intracameral delivery of anti-CTGF small interfering RNA (siRNA) by using nanoparticles coated by hyaluronan succeeded to penetrate deeply in the outflow region and showed binding of hyaluronan to the CD44 receptors, which were overexpressed in glaucomatous eyes [60]. Hyaluronan-coated nanoparticles combined with RNA interference may provide a potential strategy for glaucoma therapy.

#### 4.1.4. Adenosine

Adenosine and several adenosine derivatives increase or decrease IOP via modulation of G-protein-coupled receptors. There are four adenosine receptors subtypes known as A1, A2a, A2B, and A3 [61]. The activation of A1 receptors in the trabecular meshwork and ciliary body reduces the outflow resistance and aqueous production and lowers IOP. Activation of A2a receptors in Schlemm's canal cells can decrease or increase IOP, whereas activation of A3 receptors increases IOP [62, 63]. Trabodenoson is an A1 receptor-selective adenosine derivative. It lowers IOP by increasing aqueous outflow through trabecular meshwork. Trabodenoson topically was well tolerated without clinically important ocular and systemic side effects. Its IOP-lowering effect was dose-dependent with a mean change of 4 mmHg from baseline at the highest dose tested [64, 65].

### 4.2. Non-IOP-Dependent Treatment Strategies: Neuroprotection

Neuroprotection strategies use signalling pathways to improve cell survival and/or prevent cell death after a pathological insult. Some of the cellular processes that result in retinal ganglion cell death and are targets of neuroprotective agents include production of external nerve-derived risk factors such as glutamate and nitric oxide (NO), deprivation of internal trophic (nutritional) factors in the nerve cells, loss of intracellular self-repair processes, or generation of intracellular destructive processes [66]. Treatment strategies can be grouped into targets that interfere with excitotoxicity, oxidative stress and mitochondrial dysfunction, inflammation-abnormal immune response, glial cell modulation, and stem cell therapy. However, any division is arbitrary as most targets are involved in several pathways and/or mechanism of action is incompletely understood. A brief overview of some promising treatment strategies is summarized.

#### 4.2.1. Excitotoxicity

Excitotoxicity refers to cell death resulting from the toxic actions of excitatory amino acids. Glutamate is the major excitatory neurotransmitter in the central nervous system. In glaucoma, pathological insult leads to elevated levels of extracellular glutamate. Sustained activation of ionotropic N-methyl-D-aspartate (NMDA) receptors by glutamate impairs cellular calcium homeostasis and activates nitric oxide synthesis, formation of free radicals, and apoptosis. In physiological conditions, Mϋller cells remove the extracellular glutamate and are neuroprotective. When the homeostasis is impaired, Mϋller cells contribute to excitotoxicity and neuronal degeneration [67]. Therapy targeting excitotoxicity has been studied for application in glaucoma.

Memantine is a noncompetitive NMDA receptor antagonist and is approved for treatment of Alzheimer's and Parkinson's disease. In animal glaucoma models, memantine protected against retinal ganglion cell loss [68, 69]. Unfortunately, in two large clinical trials, daily treatment with memantine for 4 years did not prevent or delay progression in patients with open-angle glaucoma and was no different from placebo [70]. This indicates the need for better trial design, such as selecting patients with rapid progression and more sensitive endpoint for detecting progression [70, 71].

Brimonidine, an *α*-2 adrenergic agonist, is used to lower IOP. It has been shown to reduce optic nerve damage in animal glaucoma model unrelated to IOP. Brimonidine modulates glutamate-induced toxicity through several pathways. In a clinical trial, patients with low-tension glaucoma receiving brimonidine monotherapy had lower rate of progression compared with those treated with timolol over 30 months despite similar IOP (9% vs. 30%) [23]. There was a considerable dropout rate in the brimonidine group and too short follow-up, both of which limit the conclusion [72].

#### 4.2.2. Oxidative Stress and Mitochondrial Dysfunction

Mitochondria are the main source of intracellular reactive oxygen species (ROS) formed as by-product of oxidative phosphorylation. ROS are highly reactive molecules and tightly regulated under physiological conditions. In dysfunctional mitochondria, the impaired homeostasis leads to increased production of ROS with chronic oxidative damage which contributes to cellular dysfunction and neurotoxicity. Oxidative stress refers to an imbalance between generation of ROS and the cells' ability to detoxify the reactive intermediates or repair the resulting damage. Oxidative stress has been shown to play a role in retinal ganglion cell death in glaucoma. Decreased antioxidant defence status and increased oxidative stress were found in serum of patients with POAG and pseudoexfoliation glaucoma compared to controls [73]. Tanito et al. have reported that lower systemic antioxidant capacity was associated with more severe visual field loss [74]. Thus, interventions that target elevated oxidative stress and potential mitochondrial dysfunction may prove beneficial neuroprotective treatment [75]. Several compounds with antioxidant function have been studied, especially vitamin E (*α*-tocopherol), vitamin C, and *Ginkgo biloba* as oral supplementation. A systematic review did not find evidence to support the use of nutritional substances in glaucoma; the randomized clinical trials were small and biased [76]. *Ginkgo biloba* extract, mainly composed of flavonoids, is widely used nutritional supplement for treatment of cognitive impairment. *Ginkgo biloba* increases blood flow, reduces free radical damage, and interferes with glutamate signalling [77–79]. In patients with normal-tension glaucoma, *Gingko biloba* slowed progression of visual field damage [80].

Mitochondrial dysfunction has its role in the glaucoma pathogenesis. Coenzyme Q10 (CoQ10) is a mitochondrial targeted antioxidant that plays an essential role in the normal function of the electron transport chain. CoQ10 has been reported to have neuroprotective activity in neurodegenerative diseases and cerebral ischemia [81]. In addition to its antioxidant function, CoQ10 is also reported to protect against glutamate excitotoxicity [82]. In an animal model of acute IOP rise, CoQ10 was able to reduce significantly the pathological increase of glutamate observed during reperfusion and this may contribute to the neuroprotection [83]. CoQ10 associated with vitamin E topical administration in open-angle glaucoma has shown a beneficial effect on the inner retinal function with consequent enhancement of the visual cortical responses [84]. Topical CoQ10 prevented retinal ganglion cell apoptosis and loss as assessed in vivo by Detecting Apoptotic Retinal Cells (DARC) in an animal glaucoma model [84]. Two ongoing randomized clinical trials in patients with primary open-angle glaucoma treated with IOP-lowering medications are comparing addition of CoQ10 versus placebo. One trial (Phase IV, NCT04038034) is evaluating oral supplementation of CoQ10 on the functional (electrophysiological test, visual field, and contrast sensitivity) and structural (OCT) changes. The second trial (NCT03611530) is looking at the time to progression in a larger number of open-angle glaucoma patients treated with eye drops containing both CoQ10 and vitamin E versus placebo [85].

Citicoline or cytidine 5′-diphosphocholine functions as an intermediate in the membrane phospholipids. Citicoline has shown neuroprotective effects in neurodegenerative diseases, after stroke, and in cognitive impairment, brain trauma, amblyopia, and glaucoma [86]. Its mechanism of action is not clarified. Neuroprotection of retinal ganglion cells may include mimicking neurotrophic factors, reducing oxidative stress, improving axonal transport, and inhibiting excitotoxicity in retinal tissues [87–90]. Oral and intramuscular citicoline treatment used as an adjunct to IOP-lowering therapy in glaucoma patients improved pattern electroretinogram (PERG) and visually evoked potentials (VEP) and better preserved visual field compared to the placebo treated group [91–94]. Similar effects, i.e., enhanced PERG and VEP responses, in patients with glaucoma were achieved with topical citicoline therapy [95]. Recently, a clinical trial looking at the difference in glaucoma progression between the citicoline eye drop group versus the placebo group has been completed, but the results have not been published. The ongoing trial (NCT04046809) has the aim to test whether the intake of citicoline oral solution (Neurotidine®, Omikron Italia S.r.l.) can improve quality of life in patients with glaucoma.

#### 4.2.3. Inflammation-Abnormal Immune Response (Autoimmunity)

Degeneration of retinal ganglion cells and axons following pathological insult is associated with activation of microglial cells which release proinflammatory cytokines such as tumour necrosis factor-*α* (TNF-*α*) and interleukin-1*β* (IL-1*β*) [96]. Higher levels of TNF-*α* were detected in aqueous humour of patients with glaucoma compared to controls [97]. Its binding to the TNF-receptor-1 (TNF-R1) mediates retinal ganglion cell death in glaucoma [98]. Production and release of TNF-*α* occur very early following exposure to stress. In an animal model, intravitreal injection of TNF-*α* was found to induce axonal degeneration from two weeks to two months after injection, whereas significant retinal ganglion cell loss was noted at two months after injection. This effect of TNF-*α* is mediated through nuclear factor (NF)-kappa B p65 [99]. TNF-*α* can also act as a downstream mediator of proapoptotic factors such as pro-nerve growth factor (pro NGF) [100]. The finding that retinal ganglion cell apoptosis was attenuated by a neutralizing antibody against TNF-*α* supports TNF-*α* as an attractive therapeutic target [101]. The usefulness of anti TNF-*α* therapy in glaucoma will depend upon its ability to block selectively excessive TNF-*α* and TNF-R1 expression without significantly affecting its physiological functions such as local immunity.

Several studies have shown difference in the concentrations of autoantibodies in serum and aqueous humour of patients with glaucoma compared to controls. Autoantibodies changes detected include elevated levels of antibodies against *α*-fodrin, glutathione-S-transferase, spectrin, and Heat Shock Protein (HSP) 70 and decreased levels of antibodies against *α*B crystalline, vimentin, Glial Fibrillary Acidic Protein (GFAP), and ϒ-Synuclein [102–105]. *In vitro* studies have shown that antibodies against ϒ-Synuclein and GFAP possess direct and indirect (through Mϋller cells) protective effect on the retinal ganglion cells [106]. Results from clinical studies revealed altered immunoreactivities against retina and optic nerve in sera and aqueous humour of glaucoma patients which indicates a role of autoimmunity in glaucomatous neurodegeneration and retinal ganglion cells death. Targeting immune changes in the retina of glaucoma patients, such as the antibody against ϒ-Synuclein, may be a promising therapeutic strategy [107].

#### 4.2.4. Glial Cell Modulation

Glial cells regulate tightly retinal ganglion cells and their response to injury is important for maintaining the health of retina or its degeneration. The glial cells include microglial cells, which are immunocompetent cells involved in the process of apoptosis and removal of dead cells, and macroglial cells. In the nonmyelinated region in the retina, the major macroglia cells are astrocytes and Mϋller cells which form blood-retina barrier, connect the neurons to the blood-vessels, and maintain homeostasis by removing ions and neurotransmitters [108]. Mϋller cells were shown to increase uptake of excitatory glutamate in limited energy supply condition thus protecting retinal ganglion cells [109]. Macroglial cells produce cytokines such as TGF-*α*, ciliary neurotrophic factor (CNTF), and platelet-derived growth factor [110–112]. CNTF is one of the most extensively studied neurotrophic factors, which was able to induce neuronal cell differentiation and neurite outgrowth and protect cells from neurodegeneration in an animal model following axotomy [113]. The CNTF concentration was reduced in aqueous humour and lacrimal fluid of patients with primary open-angle glaucoma, especially in those with severe visual loss [114]. CNTF is a promising target and its neuroprotective effect was evaluated in phase I study (NCT01408472) in patients with POAG who received intraocular implant NT-501 CNTF (made by Neurotech) into one eye. The NT-501 implant contains encapsulated retinal pigment epithelial cells that have been modified to release CNTF across a semipermeable membrane in a selective and sustained way. The results of the study have not been published. Another ongoing phase 2 trial (NCT02862938) is evaluating safety and efficacy of NT-501 CNTF intravitreal implant versus sham surgery.

Intravitreal injection of platelet-derived growth factor (PDGF) inhibited retinal ganglion cell death. The neuroprotective effect of PDGF has been shown to be mediated by astrocytes and amacrine cells which are in the presence of PDGF stimulated to secrete factors protecting ganglion cells [115, 116]. Therefore, modulation of macroglial cell activity has a potential in neuroprotection.

#### 4.2.5. Stem Cell Therapy

Regarding the origin, stem cells can be divided into embryonic, adult, and induced pluripotent stem cells. The latter are artificially produced from any somatic cell by reprogramming its properties into a pluripotent stem cell. Among the adult stem cells, mesenchymal stromal cells (MSC) have been shown to be neuroprotective and promote regeneration in an animal glaucoma model and after optic nerve injury [117–119]. Therapeutic effects of MSC are mediated by their immunomodulatory and secretory properties, production of numerous cytokines, and growth factors. MSC can also differentiate into different cell types [120]. It has been shown in ex vivo human retinal explants that PDGF plays an important role in MSC-mediated retinal ganglion cell protection and may represent a new target in retinal ganglion cell neuroprotection [121, 122]. The spectrum and concentration of immunoregulatory molecules produced by the MSC depend on the environment [123]. The side effects of MSC following intravitreal administration have been reported and may be influenced by the difference of diseased environment, indications, and inconsistencies in isolation and preparation of MSC [124–126]. Two ongoing trials (NCT01920867; NCT03011541) aim to evaluate autologous bone marrow-derived MSC for treatment of multiple retinal diseases including glaucoma. A completed phase 1 study evaluating intravitreal application of autologous bone marrow-derived MSC in advanced glaucoma (NCT02330978) enrolled 2 patients. One patient developed retinal detachment with proliferative vitreoretinopathy and lost light perception. Study using autologous adipose tissue-derived stem cells delivered sub-Tenon's in glaucoma patients is going on in Russia (NCT02144103). These trials are mainly focused on safety, followed by efficacy, and are designed to determine the best method of delivery and the required level of immunosuppression [127]. Stem cell therapy has a potential for glaucoma treatment and needs further evaluation in well-designed clinical studies.

#### 4.2.6. Gene Therapy

Gene therapy aims to correct a specific, well defined genetic defect or deliver protective factors using different pathways to stimulate survival and regeneration of retinal ganglion cells. The most promising vector systems for successful gene delivery in the eye are recombinant adenoassociated viral vectors (AAVs), which lead to long and sustained levels of gene expression within a select target cell [128]. Genetic approach is still in preclinical phase for glaucoma. Correcting a specific genetic defect is feasible in primary congenital or primary juvenile open-angle glaucoma, both of which have a clear genetic basis. Between 10 and 30% of patients with primary juvenile open-angle glaucoma have mutations in the gene encoding myocilin that affects trabecular meshwork function with an increase in IOP [129, 130]. Recently, Jain et al. have disrupted the effects of the mutant myocilin gene using AAV-CRISP/Cas9 in a mouse model of myocilin-associated glaucoma and were able to lower IOP and prevent further glaucomatous damage [131]. The etiopathogenesis of adult-onset glaucoma is not clear and includes various genetic, environmental, and individual risk factors. For these reasons, gene therapy strategies are based on enhancing retinal ganglion cell survival or inhibiting cell death pathways [128]. Supplementation of brain-derived neurotrophic factors showed transient neuroprotective effect due to BDNF receptor (TrKB) downregulation [132]. A novel AAV gene therapy (AAV2 TrKB-2A-mBDNF) increased production of BDNF and the expression of its receptor. The neuroprotective efficacy was confirmed in an experimental animal model of glaucoma and optic nerve injury and was present over 6 months without vector-related adverse effects [133]. Although there are major advances in gene therapy such as in Leber's hereditary optic neuropathy, in adult-onset glaucoma there are many unresolved issues such as which molecular pathways to be targeted, long-term modification of gene expression, and immunogenic and mutagenic effects [129]. Gene therapy is a promising treatment strategy for neuroprotection, but further research and studies are needed.

## 5. Future Trends in Glaucoma Therapy

### 5.1. Sustained Drug Delivery Systems: Sustained Release Drug Formulations

Poor adherence is an important issue in the long-term glaucoma therapy. To avoid active instillation of eye drops, several sustained drug delivery systems have been developed.

Bimatoprost SR (Allergan, Dublin, Ireland) is a biodegradable implant which is injected in the anterior chamber and enables a slow, extended release of medication. In phase I/II, bimatoprost SR was safe and showed comparable efficacy to topical bimatoprost through 6 months [134]. The side effects of bimatoprost SR included conjunctival hyperaemia, foreign body sensation, punctate keratitis, increased lacrimation, conjunctival haemorrhage, eye pain, transient iritis, and progression of cataracts [134]. Bimatoprost SR is currently in six phase III studies. Two studies (NCT 02636946; NCT02507687) are comparing efficacy and safety of bimatoprost SR to selective laser trabeculoplasty, 3 studies aim to assess long-term efficacy and safety of bimatoprost SR (NCT03850782; NCT 03891446; NCT02250651), and one completed study (NCT02247804) compared safety and efficacy of bimatoprost SR to topical timolol bid. The results have not been published yet.

The topical bimatoprost ocular insert (Allergan, Dublin, Ireland) is an ocular ring which contains 13 mg of bimatoprost incorporated within a silicone matrix with an inner polypropylene structure. The ring is inserted between the upper and lower fornix. It releases drug into the tear film in a decreasing concentration over six months. Bimatoprost ring lowered IOP by 3.2–6.4 mmHg from baseline IOP and was noninferior to topical timolol [135]. The ring was safe and well tolerated and stayed in place in 95% of subjects [136]. There is no data about potential availability of this delivery system on the market.

Currently there are 3 ongoing trials evaluating a titanium intraocular implant filled with travoprost (Glaukos, Inc.) that releases travoprost with two different elution rates (NCT02754596; NCT03868124; NCT03519386) and comparing it to topical timolol treatment.

Travoprost extended release as a biodegradable intracameral implant (Envisia Therapeutics) has been evaluated in a phase II study for up to 24 months. Ocular side effects included ocular hyperaemia, photophobia, anterior chamber inflammation (iritis), cataract, and corneal endothelial cell loss (NCT02371746).

Intracanalicular insert of sustained release travoprost OTX-TP (Ocular Therapeutix, Inc.) is a hydrogel punctum plug eluting drug into the tear film. Completed phase III study (NCT02914509) has not published results yet. Currently an ongoing open-label phase III study is evaluating long-term safety of repeat dose punctum plug delivery over 12 months.

Recently, the micelles-laden contact lenses have been developed and were able to achieve sustained release of timolol and latanoprost simultaneously [137].

The development in sustained drug release is promising, but there are still unsolved issues, such as long-term safety with intraocular implants compared to eye drops, variation in the length of time of IOP-lowering effect, and costs. Also, approximately half of patients require more than one drug for IOP control and development of delivery systems with simultaneous release of more than one drug with different properties to avoid instillation of eye drops is still a challenge.

At present, there is no solid evidence that topical or systemic neuroprotective agents and nutritional supplements may be beneficial for individuals with open-angle glaucoma and IOP-lowering eye drops remain the only proven and available treatment for glaucoma [72, 76]. Adherence represents a treatment burden and it has been reported that 60% of patients had one or more problems with taking their medication [138]. One-third up to 75% of glaucoma patients do not use their eye drops as prescribed [139, 140]. Lower adherence has been reported with younger age, male gender, forgetfulness, lower social status and education, medication cost, side effects, greater number of daily instillations, and situational obstacles (travel and change of daily routine) [139, 141–143]. In the longitudinal assessment of patients in the Collaborative Initial Glaucoma Treatment Study lower adherence was associated with faster visual field loss [144]. A systematic review assessing different interventions to improve patients' adherence to topical glaucoma therapy found that there was insufficient evidence to recommend any interventions to improve adherence, but simplified drug regimens could be of benefit [145].

In the near future, the sustained drug-release implants and nanotechnology based-drugs for glaucoma using nano delivery systems have a potential to overcome the limitations of topical IOP-lowering drops by improving bioavailability, providing sustained release, targeted delivery, dose accuracy, and reducing side effects [146, 147].

### 5.2. Personalised Medicine and Biomarkers of Disease

Personalised medicine refers to tailoring glaucoma prevention and treatment individually based on genetic and other characteristics of the individual patient. Most of the open-angle glaucoma forms are complex and have polygenic basis resulting from a combined effect of several common gene variants, each of which has a small effect size on the disease [148]. Genome-wide association analyses have identified several loci associated with glaucoma risk factors such as IOP, vertical cup-disc ratio, and central corneal thickness [149–151]. The genetic findings need to be integrated with risk factors to identify patients at high risk of progression to visual impairment [152]. Using the large data set machine learning can cluster patients based on their genomic similarity and detect relevant pathways that are disrupted in glaucoma [153]. Investigation of these pathways can detect new biomarkers for glaucoma diagnosis, prognosis, and new therapeutic targets, which after validation will help ophthalmologists to identify patients with high risk of progression and treat them more aggressively and avoid unnecessary treatment to many subjects [154].

Many different measurable indicators can serve as potential biomarkers for glaucoma such as IOP or OCT measurements of retinal nerve fibre layer thickness [155]. With the advances in technology including imaging, genomic, metabolomic, and proteomic techniques, potential new biomarkers are generated and need to be validated in large patients' populations with different ethnicity and stage of glaucoma [156, 157]. These biomarkers may serve as diagnostic, predictive, prognostic biomarkers or indicate patients' response to drug or surgery [158, 159]. Recently, aqueous veins were found to be a potential structural biomarker predicting the outcome of Schlemm's canal-based glaucoma surgery [160].

## 6. Conclusions

The present review summarizes current treatment strategies in glaucoma therapy and addresses potential future targets and ways to protect and improve survival and regeneration of retinal ganglion cells. Targeting several pathways has been shown to improve survival of retinal ganglion cells in animal glaucoma models. However, translation to the clinic is hampered due to the limitations of glaucoma models and the fact that glaucoma pathogenesis is multifactorial and incompletely understood. Further research is required to identify molecules and pathways to be able to improve clinical translation of neuroprotection in glaucoma. Currently lowering of IOP remains the only treatment strategy and adherence to treatment is essential. Sustained drug delivery systems aim to overcome adherence problem, but there are still unresolved issues with safety, duration of IOP-lowering effect, treatment with several compounds simultaneously, quality of life, and costs.

For future personalized medicine that considers individual variability in genes, environmental and lifestyle factors for each person hold a promise to predict optimal treatment and prevention strategies for the individual glaucoma patient.

## Figures and Tables

**Table 1:** IOP-lowering medications, efficacy, and mechanism of action.

Medication Class	Compound	IOP reduction (%)	Mechanism of action			
			Increases uveoscleral outflow	Increases trabecular outflow	Decreases aqueous production	Decreases episcleral venous pressure
*Prostaglandin analogues*	Bimatoprost, latanoprost, tafluprost, travoprost	25–35	Yes	No	No	No
*β-Blockers* (i) Nonselective	Timolol, levobonolol, carteolol, metipranolol	20–25	No	No	Yes	No
(ii) *β*1-Selective	Betaxolol	20	No	No	Yes	No
*Carbonic anhydrase inhibitors* (i) *Topical*	Dorzolamide, brinzolamide	20	No	No	Yes	No
(ii) *Systemic*	Acetazolamide, methazolamide, dichlorphenamide	30–40	No	No	Yes	No
*Adrenergic agonists* (i) *α*-2 Selective	Brimonidine, apraclonidine	20–25	Yes	No	Yes	No
(ii) Nonselective	Dipivefrin, epinephrine	15–20	Yes	No	Yes	No
*Parasympathomimetics*	Pilocarpine, echothiophate	20–25	No	Yes	No	No

*Novel IOP-lowering medications*						
*Rho-kinase inhibitors*	Netarsudil	16–21	No	Yes	Yes	Yes
*Nitric oxide-donating prostaglandin analogue*	Latanoprostene bunod	32–34	Yes	Yes	No	No
FC rho-kinase inhibitor/latanoprost	Netarsudil/latanoprost	30–36	Yes	Yes	Yes	Yes

## References

[1] Flaxman S. R., Bourne R. R. A., Resnikoff S. (2017). Global causes of blindness and distance vision impairment 1990–2020: a systematic review and meta-analysis. *The Lancet Global Health*.

[2] Tham Y.-C., Li X., Wong T. Y., Quigley H. A., Aung T., Cheng C.-Y. (2014). Global prevalence of glaucoma and projections of glaucoma burden through 2040: a systematic review and meta-analysis. *Ophthalmology*.

[3] Peters D., Bengtsson B., Heijl A. (2013). Lifetime risk of blindness in open-angle glaucoma. *American Journal of Ophthalmology*.

[4] Yadav K. S., Rajpurohit R., Sharma S. (2019). Glaucoma: current treatment and impact of advanced drug delivery systems. *Life Sciences*.

[5] Mozaffarieh M., Flammer J. (2013). New insights in the pathogenesis and treatment of normal tension glaucoma. *Current Opinion in Pharmacology*.

[6] Adeghate J., Rahmatnejad K., Waisbourd M., Katz L. J. (2019). Intraocular pressure-independent management of normal tension glaucoma. *Survey of Ophthalmology*.

[7] Kass M. A., Heuer D. K., Higginbotham E. J. (2002). The ocular hypertension treatment study: a randomized trial determines that topical ocular hypotensive medication delays or prevents the onset of primary open-angle glaucoma. *Archives of Ophthalmology*.

[8] Heijl A., Leske M. C., Bengtsson B., Hyman L., Bengtsson B., Hussein M. (2002). Reduction of intraocular pressure and glaucoma progression: results from the early manifest glaucoma trial. *Archives of Ophthalmology*.

[9] The Advanced Glaucoma Intervention Study (AGIS): 7 (2000). The relationship between control of intraocular pressure and visual field deterioration. The AGIS investigators. *American Journal of Ophthalmology*.

[10] Li T., Lindsley K., Rouse B. (2016). Comparative effectiveness of first-line medications for primary open-angle glaucoma: a systematic review and network meta-analysis. *Ophthalmology*.

[11] Li F., Huang W., Zhang X. (2018). Efficacy and safety of different regimens for primary open-angle glaucoma or ocular hypertension: a systematic review and network meta-analysis. *Acta Ophthalmologica*.

[12] Aptel F., Cucherat M., Denis P. (2008). Efficacy and tolerability of prostaglandin analogs: a meta-analysis of randomized controlled clinical trials. *Journal of Glaucoma*.

[13] Camras C. B., Alm A., Watson P., Stjernschantz J. (1996). Latanoprost, a prostaglandin analog, for glaucoma therapy. Efficacy and safety after 1 year of treatment in 198 patients. Latanoprost study groups. *Ophthalmology*.

[14] Inoue K., Shiokawa M., Higa R. (2012). Adverse periocular reactions to five types of prostaglandin analogs. *Eye*.

[15] Wand M., Gilbert C. M., Liesegang T. J. (1999). Latanoprost and herpes simplex keratitis. *American Journal of Ophthalmology*.

[16] Coakes R. L., Brubaker R. F. (1978). The mechanism of timolol in lowering intraocular pressure. In the normal eye. *Archives of Ophthalmology*.

[17] Taniguchi T., Kitazawa Y. (1997). The potential systemic effect of topically applied *β*-blockers in glaucoma therapy. *Current Opinion in Ophthalmology*.

[18] Caldwell D. R., Salisbury C. R., Guzek J. P. (1984). Effects of topical betaxolol in ocular hypertensive patients. *Archives of Ophthalmology*.

[19] Marquis R. E., Whitson J. T. (2005). Management of glaucoma: focus on pharmacological therapy. *Drugs & Aging*.

[20] Konowal A., Morrison J. C., Brown S. V. L. (1999). Irreversible corneal decompensation in patients treated with topical dorzolamide. *American Journal of Ophthalmology*.

[21] Schuman J. S., Horwitz B., Choplin N. T., David R., Albracht D., Chen K. (1997). A 1-year study of brimonidine twice daily in glaucoma and ocular hypertension. A controlled, randomized, multicenter clinical trial. Chronic brimonidine study group. *Archives of Ophthalmology*.

[22] Katz L. J. (1999). Brimonidine tartrate 0.2% twice daily vs timolol 0.5% twice daily: 1-year results in glaucoma patients. Brimonidine study group. *American Journal of Ophthalmology*.

[23] Krupin T., Liebmann J. M., Greenfield D. S., Ritch R., Gardiner S., Low-Pressure Glaucoma Study G. (2011). A randomized trial of brimonidine versus timolol in preserving visual function: results from the low-pressure glaucoma treatment study. *American Journal of Ophthalmology*.

[24] Aerie Pharmaceuticals (2019). Aerie pharmaceuticals receives positive CHMP opinion for Rhokiinsa in the European Union. https://eyewire.news/articles/aerie-pharmaceuticals-receives-positive-chmp-opinion-for-rhokiinsa-in-the-european-union/.

[25] Tanna A. P., Johnson M. (2018). Rho kinase inhibitors as a novel treatment for glaucoma and ocular hypertension. *Ophthalmology*.

[26] Bacharach J., Dubiner H. B., Levy B., Kopczynski C. C., Novack G. D., Group A.-C. S. (2015). Double-masked, randomized, dose-response study of AR-13324 versus latanoprost in patients with elevated intraocular pressure. *Ophthalmology*.

[27] Serle J. B., Katz L. J., McLaurin E. (2018). Two phase 3 clinical trials comparing the safety and efficacy of netarsudil to timolol in patients with elevated intraocular pressure: rho kinase elevated IOP treatment trial 1 and 2 (ROCKET-1 and ROCKET-2). *American Journal of Ophthalmology*.

[28] Kahook M. Y., Serle J. B., Mah F. S. (2019). Long-term safety and ocular hypotensive efficacy evaluation of netarsudil ophthalmic solution: rho kinase elevated IOP treatment trial (ROCKET-2). *American Journal of Ophthalmology*.

[29] Khouri A. S., Serle J. B., Bacharach J. (2019). Once-daily netarsudil versus twice-daily timolol in patients with elevated intraocular pressure: the randomized phase 3 ROCKET-4 study. *American Journal of Ophthalmology*.

[30] Cavet M. E., Vittitow J. L., Impagnatiello F., Ongini E., Bastia E. (2014). Nitric oxide (NO): an emerging target for the treatment of glaucoma. *Investigative Opthalmology & Visual Science*.

[31] Goldstein I. M., Ostwald P., Roth S. (1996). Nitric oxide: a review of its role in retinal function and disease. *Vision Research*.

[32] Schmetterer L., Polak K. (2001). Role of nitric oxide in the control of ocular blood flow. *Progress in Retinal and Eye Research*.

[33] Brown G. C., Bal-Price A. (2003). Inflammatory neurodegeneration mediated by nitric oxide, glutamate, and mitochondria. *Molecular Neurobiology*.

[34] Thomas D. D., Liu X., Kantrow S. P., Lancaster J. R. (2001). The biological lifetime of nitric oxide: implications for the perivascular dynamics of NO and O_2_. *Proceedings of the National Academy of Sciences*.

[35] Weinreb R. N., Scassellati Sforzolini B., Vittitow J., Liebmann J. (2016). Latanoprostene bunod 0.024% versus timolol maleate 0.5% in subjects with open-angle glaucoma or ocular hypertension: the APOLLO study. *Ophthalmology*.

[36] Medeiros F. A., Martin K. R., Peace J., Scassellati Sforzolini B., Vittitow J. L., Weinreb R. N. (2016). Comparison of latanoprostene bunod 0.024% and timolol maleate 0.5% in open-angle glaucoma or ocular hypertension: the LUNAR study. *American Journal of Ophthalmology*.

[37] Weinreb R. N., Liebmann J. M., Martin K. R., Kaufman P. L., Vittitow J. L. (2018). Latanoprostene bunod 0.024% in subjects with open-angle glaucoma or ocular hypertension: pooled phase 3 study findings. *Journal of Glaucoma*.

[38] Kaufman P. L. (2017). Latanoprostene bunod ophthalmic solution 0.024% for IOP lowering in glaucoma and ocular hypertension. *Expert Opinion on Pharmacotherapy*.

[39] Kawase K., Vittitow J. L., Weinreb R. N., Araie M., Group J. S. (2016). Long-term safety and efficacy of latanoprostene bunod 0.024% in Japanese subjects with open-angle glaucoma or ocular hypertension: the JUPITER study. *Advances in Therapy*.

[40] Aerie Pharmaceuticals (2019). Aerie pharmaceuticals receives FDA approval of rocklatan for reduction of IOP. https://eyewire.news/articles/aerie-pharmaceuticals-receives-fda-approval-of-rocklatan-for-reduction-of-iop/.

[41] Asrani S., Robin A. L., Serle J. B. (2019). Netarsudil/latanoprost fixed-dose combination for elevated intraocular pressure: three-month data from a randomized phase 3 trial. *American Journal of Ophthalmology*.

[42] Walters T. R., Ahmed I. I. K., Lewis R. A. (2019). Once-daily netarsudil/Latanoprost fixed-dose combination for elevated intraocular pressure in the randomized phase 3 MERCURY-2 study. *Ophthalmology Glaucoma*.

[43] Sugrue M. F. (1997). New approaches to antiglaucoma therapy. *Journal of Medicinal Chemistry*.

[44] Cairns E. A., Baldridge W. H., Kelly M. E. (2016). The endocannabinoid system as a therapeutic target in glaucoma. *Neural Plasticity*.

[45] Rapino C., Tortolani D., Scipioni L., Maccarrone M. (2018). Neuroprotection by (endo)cannabinoids in glaucoma and retinal neurodegenerative diseases. *Current Neuropharmacology*.

[46] Porcella A., Maxia C., Gessa G. L., Pani L. (2001). The synthetic cannabinoid WIN55212-2 decreases the intraocular pressure in human glaucoma resistant to conventional therapies. *European Journal of Neuroscience*.

[47] Crandall J., Matragoon S., Khalifa Y. M. (2007). Neuroprotective and intraocular pressure-lowering effects of (−)Δ^9^-tetrahydrocannabinol in a rat model of glaucoma. *Ophthalmic Research*.

[48] El-Remessy A. B., Khalil I. E., Matragoon S. (2003). Neuroprotective effect of (−)Δ^9^-tetrahydrocannabinol and cannabidiol in N-methyl-D-aspartate-induced retinal neurotoxicity: involvement of peroxynitrite. *The American Journal of Pathology*.

[49] Whiting P. F., Wolff R. F., Deshpande S. (2015). Cannabinoids for medical use: a systematic review and meta-analysis. *JAMA*.

[50] Taskar P. S., Patil A., Lakhani P. (2019). Delta(9)-tetrahydrocannabinol derivative-loaded nanoformulation lowers intraocular pressure in normotensive rabbits. *Translational Vision Science & Technology*.

[51] Alkozi H. A., Navarro G., Franco R., Pintor J. (2019). Melatonin and the control of intraocular pressure. *Progress in Retinal and Eye Research*.

[52] Alarma-Estrany P., Guzman-Aranguez A., Huete F. (2011). Design of novel melatonin analogs for the reduction of intraocular pressure in normotensive rabbits. *Journal of Pharmacology and Experimental Therapeutics*.

[53] Martinez-Aguila A., Fonseca B., Perez de Lara M. J., Pintor J. (2016). Effect of melatonin and 5-methoxycarbonylamino-N-acetyltryptamine on the intraocular pressure of normal and glaucomatous mice. *Journal of Pharmacology and Experimental Therapeutics*.

[54] Pescosolido N., Gatto V., Stefanucci A., Rusciano D. (2015). Oral treatment with the melatonin agonist agomelatine lowers the intraocular pressure of glaucoma patients. *Ophthalmic and Physiological Optics*.

[55] Lundmark P. O., Pandi-Perumal S. R., Srinivasan V., Cardinali D. P., Rosenstein R. E. (2007). Melatonin in the eye: implications for glaucoma. *Experimental Eye Research*.

[56] Fuchshofer R., Tamm E. R. (2009). Modulation of extracellular matrix turnover in the trabecular meshwork. *Experimental Eye Research*.

[57] Browne J. G., Ho S. L., Kane R. (2011). Connective tissue growth factor is increased in pseudoexfoliation glaucoma. *Investigative Ophthalmology & Visual Science*.

[58] Junglas B., Yu A. H., Welge-Lussen U., Tamm E. R., Fuchshofer R. (2009). Connective tissue growth factor induces extracellular matrix deposition in human trabecular meshwork cells. *Experimental Eye Research*.

[59] Junglas B., Kuespert S., Seleem A. A. (2012). Connective tissue growth factor causes glaucoma by modifying the actin cytoskeleton of the trabecular meshwork. *American Journal of Pathology*.

[60] Dillinger A. E., Guter M., Froemel F. (2018). Intracameral delivery of layer-by-layer coated siRNA nanoparticles for glaucoma therapy. *Small*.

[61] Guglielmi P., Carradori S., Campestre C., Poce G. (2019). Novel therapies for glaucoma: a patent review (2013–2019). *Expert Opinion on Therapeutic Patents*.

[62] Zhong Y., Yang Z., Huang W. C., Luo X. (2013). Adenosine, adenosine receptors and glaucoma: an updated overview. *Biochimica et Biophysica Acta*.

[63] Shearer T. W., Crosson C. E. (2002). Adenosine A1 receptor modulation of MMP-2 secretion by trabecular meshwork cells. *Investigative Ophthalmology & Visual Science*.

[64] Laties A., Rich C. C., Stoltz R. (2016). A randomized phase 1 dose escalation study to evaluate safety, tolerability, and pharmacokinetics of trabodenoson in healthy adult volunteers. *Journal of Ocular Pharmacology and Therapeutics: The Official Journal of the Association for Ocular Pharmacology and Therapeutics*.

[65] Myers J. S., Sall K. N., DuBiner H. (2016). A dose-escalation study to evaluate the safety, tolerability, pharmacokinetics, and efficacy of 2 and 4 weeks of twice-daily ocular trabodenoson in adults with ocular hypertension or primary open-angle glaucoma. *Journal of Ocular Pharmacology and Therapeutics: The Official Journal of the Association for Ocular Pharmacology and Therapeutics*.

[66] Schwartz M., Yoles E. (2000). Neuroprotection: a new treatment modality for glaucoma?. *Current Opinion in Ophthalmology*.

[67] Furuya T., Pan Z., Kashiwagi K. (2012). Role of retinal glial cell glutamate transporters in retinal ganglion cell survival following stimulation of NMDA receptor. *Current Eye Research*.

[68] Hare W. A., WoldeMussie E., Lai R. K. (2004). Efficacy and safety of memantine treatment for reduction of changes associated with experimental glaucoma in monkey, I: functional measures. *Investigative Ophthalmology & Visual Science*.

[69] Yucel Y. H., Gupta N., Zhang Q., Mizisin A. P., Kalichman M. W., Weinreb R. N. (2006). Memantine protects neurons from shrinkage in the lateral geniculate nucleus in experimental glaucoma. *Archives of Ophthalmology*.

[70] Weinreb R. N., Liebmann J. M., Cioffi G. A. (2018). Oral memantine for the treatment of glaucoma: design and results of 2 randomized, placebo-controlled, phase 3 studies. *Ophthalmology*.

[71] Osborne N. N. (2009). Recent clinical findings with memantine should not mean that the idea of neuroprotection in glaucoma is abandoned. *Acta Ophthalmologica*.

[72] Sena D. F., Lindsley K. (2017). Neuroprotection for treatment of glaucoma in adults. *Cochrane Database Systematic Review*.

[73] Erdurmus M., Yagci R., Atis O., Karadag R., Akbas A., Hepsen I. F. (2011). Antioxidant status and oxidative stress in primary open angle glaucoma and pseudoexfoliative glaucoma. *Current Eye Research*.

[74] Tanito M., Kaidzu S., Takai Y., Ohira A. (2016). Association between systemic oxidative stress and visual field damage in open-angle glaucoma. *Scientific Reports*.

[75] Lopez Sanchez M. I., Crowston J. G., Mackey D. A., Trounce I. A. (2016). Emerging mitochondrial therapeutic targets in optic neuropathies. *Pharmacology & Therapeutics*.

[76] Loskutova E., O’Brien C., Loskutov I., Loughman J. (2019). Nutritional supplementation in the treatment of glaucoma: a systematic review. *Survey of Ophthalmology*.

[77] Janssens D., Michiels C., Delaive E., Eliaers F., Drieu K., Remacle J. (1995). Protection of hypoxia-induced ATP decrease in endothelial cells by Ginkgo biloba extract and bilobalide. *Biochemical Pharmacology*.

[78] Abdel-Kader R., Hauptmann S., Keil U. (2007). Stabilization of mitochondrial function by Ginkgo biloba extract (EGb 761). *Pharmacological Research*.

[79] Eckert A., Keil U., Scherping I., Hauptmann S., Muller W. E. (2005). Stabilization of mitochondrial membrane potential and improvement of neuronal energy metabolism by Ginkgo biloba extract EGb 761. *Annals of the New York Academy of Sciences*.

[80] Lee J., Sohn S. W., Kee C. (2013). Effect of Ginkgo biloba extract on visual field progression in normal tension glaucoma. *Journal of Glaucoma*.

[81] Ahmed E., Donovan T., Yujiao L., Zhang Q. (2015). Mitochondrial targeted antioxidant in cerebral ischemia. *Journal of Neurology and Neuroscience*.

[82] Papucci L., Schiavone N., Witort E. (2003). Coenzyme q10 prevents apoptosis by inhibiting mitochondrial depolarization independently of its free radical scavenging property. *Journal of Biological Chemistry*.

[83] Russo R., Cavaliere F., Rombola L. (2008). Rational basis for the development of coenzyme Q10 as a neurotherapeutic agent for retinal protection. *Progress in Brain Research*.

[84] Parisi V., Centofanti M., Gandolfi S. (2014). Effects of coenzyme Q10 in conjunction with vitamin E on retinal-evoked and cortical-evoked responses in patients with open-angle glaucoma. *Journal of Glaucoma*.

[85] Quaranta L., Riva I., Biagioli E. (2019). Evaluating the effects of an ophthalmic solution of coenzyme Q10 and vitamin E in open-angle glaucoma patients: a study protocol. *Advances in Therapy*.

[86] (2008). Citicoline. Monograph. *Alternative Medicine Review*.

[87] Oshitari T., Yoshida-Hata N., Yamamoto S. (2010). Effect of neurotrophic factors on neuronal apoptosis and neurite regeneration in cultured rat retinas exposed to high glucose. *Brain Research*.

[88] Matteucci A., Varano M., Gaddini L. (2014). Neuroprotective effects of citicoline in in vitro models of retinal neurodegeneration. *International Journal of Molecular Sciences*.

[89] Grieb P., Junemann A., Rekas M., Rejdak R. (2016). Citicoline: a food beneficial for patients suffering from or threated with glaucoma. *Frontiers in Aging Neuroscience*.

[90] Qian K., Gu Y., Zhao Y., Li Z., Sun M. (2014). Citicoline protects brain against closed head injury in rats through suppressing oxidative stress and calpain over-activation. *Neurochemical Research*.

[91] Virno M., Pecori-Giraldi J., Liguori A., Gregorio F. (2000). The protective effect of citicoline on the progression of the perimetric defects in glaucomatous patients (perimetric study with a 10-year follow-up). *Acta Ophthalmologica Scandinavica*.

[92] Parisi V. (2005). Electrophysiological assessment of glaucomatous visual dysfunction during treatment with cytidine-5′-diphosphocholine (citicoline): a study of 8 years of follow-up. *Documenta Ophthalmologica*.

[93] Ottobelli L., Manni G. L., Centofanti M., Iester M., Allevena F., Rossetti L. (2013). Citicoline oral solution in glaucoma: is there a role in slowing disease progression?. *Ophthalmologica*.

[94] Lanza M., Gironi Carnevale U. A., Mele L., Bifani Sconocchia M., Bartollino S., Costagliola C. (2019). Morphological and functional evaluation of oral citicoline therapy in chronic open-angle glaucoma patients: a pilot study with a 2-year follow-up. *Frontiers in Pharmacology*.

[95] Parisi V., Centofanti M., Ziccardi L. (2015). Treatment with citicoline eye drops enhances retinal function and neural conduction along the visual pathways in open angle glaucoma. *Graefe’s Archive for Clinical and Experimental Ophthalmology*.

[96] Osborne N. N. (2010). Mitochondria: their role in ganglion cell death and survival in primary open angle glaucoma. *Experimental Eye Research*.

[97] Sawada H., Fukuchi T., Tanaka T., Abe H. (2010). Tumor necrosis factor-alpha concentrations in the aqueous humor of patients with glaucoma. *Investigative Ophthalmology & Visual Science*.

[98] Tezel G., Li L. Y., Patil R. V., Wax M. B. (2001). TNF-alpha and TNF-alpha receptor-1 in the retina of normal and glaucomatous eyes. *Investigative Ophthalmology & Visual Science*.

[99] Kitaoka Y., Kitaoka Y., Kwong J. M. (2006). TNF-alpha-induced optic nerve degeneration and nuclear factor-kappaB p65. *Investigative Ophthalmology & Visual Science*.

[100] Al-Gayyar M. M., Matragoon S., Pillai B. A., Ali T. K., Abdelsaid M. A., El-Remessy A. B. (2011). Epicatechin blocks pro-nerve growth factor (proNGF)-mediated retinal neurodegeneration via inhibition of p75 neurotrophin receptor expression in a rat model of diabetes [corrected]. *Diabetologia*.

[101] Tezel G., Wax M. B. (2000). Increased production of tumor necrosis factor-alpha by glial cells exposed to simulated ischemia or elevated hydrostatic pressure induces apoptosis in cocultured retinal ganglion cells. *The Journal of Neuroscience*.

[102] Tezel G., Seigel G. M., Wax M. B. (1998). Autoantibodies to small heat shock proteins in glaucoma. *Investigative Ophthalmology & Visual Science*.

[103] Yang J., Tezel G., Patil R. V., Romano C., Wax M. B. (2001). Serum autoantibody against glutathione S-transferase in patients with glaucoma. *Investigative Ophthalmology & Visual Science*.

[104] Joachim S. C., Reichelt J., Berneiser S., Pfeiffer N., Grus F. H. (2008). Sera of glaucoma patients show autoantibodies against myelin basic protein and complex autoantibody profiles against human optic nerve antigens. *Graefe’s Archive for Clinical and Experimental Ophthalmology*.

[105] Joachim S. C., Bruns K., Lackner K. J., Pfeiffer N., Grus F. H. (2007). Antibodies to alpha B-crystallin, vimentin, and heat shock protein 70 in aqueous humor of patients with normal tension glaucoma and IgG antibody patterns against retinal antigen in aqueous humor. *Current Eye Research*.

[106] Bell K., Wilding C., Funke S. (2016). Neuroprotective effects of antibodies on retinal ganglion cells in an adolescent retina organ culture. *Journal of Neurochemistry*.

[107] Bell K., Funke S., Grus F. H. (2019). Autoimmunity and glaucoma. *Ophthalmologe*.

[108] Wang J. W., Chen S. D., Zhang X. L., Jonas J. B. (2016). Retinal microglia in glaucoma. *Journal of Glaucoma*.

[109] Toft-Kehler A. K., Skytt D. M., Poulsen K. A. (2014). Limited energy supply in Muller cells alters glutamate uptake. *Neurochemical Research*.

[110] Junier M. P. (2000). What role(s) for TGFalpha in the central nervous system?. *Progress in Neurobiology*.

[111] Liu X., Clark A. F., Wordinger R. J. (2007). Expression of ciliary neurotrophic factor (CNTF) and its tripartite receptor complex by cells of the human optic nerve head. *Molecular Vision*.

[112] Gris P., Tighe A., Levin D., Sharma R., Brown A. (2007). Transcriptional regulation of scar gene expression in primary astrocytes. *Glia*.

[113] Pasquin S., Sharma M., Gauchat J. F. (2015). Ciliary neurotrophic factor (CNTF): new facets of an old molecule for treating neurodegenerative and metabolic syndrome pathologies. *Cytokine & Growth Factor Reviews*.

[114] Shpak A. A., Guekht A. B., Druzhkova T. A., Kozlova K. I., Gulyaeva N. V. (2017). Ciliary neurotrophic factor in patients with primary open-angle glaucoma and age-related cataract. *Molecular Vision*.

[115] Chong R. S., Osborne A., Conceicao R., Martin K. R. (2016). Platelet-derived growth factor preserves retinal synapses in a rat model of ocular hypertension. *Investigative Ophthalmology & Visual Science*.

[116] Takahama S., Adetunji M. O., Zhao T., Chen S., Li W., Tomarev S. I. (2017). Retinal astrocytes and GABAergic wide-field amacrine cells express PDGFRalpha: connection to retinal ganglion cell neuroprotection by PDGF-AA. *Investigative Ophthalmology & Visual Science*.

[117] Johnson T. V., Bull N. D., Hunt D. P., Marina N., Tomarev S. I., Martin K. R. (2010). Neuroprotective effects of intravitreal mesenchymal stem cell transplantation in experimental glaucoma. *Investigative Ophthalmology & Visual Science*.

[118] Emre E., Yuksel N., Duruksu G. (2015). Neuroprotective effects of intravitreally transplanted adipose tissue and bone marrow-derived mesenchymal stem cells in an experimental ocular hypertension model. *Cytotherapy*.

[119] Mead B., Logan A., Berry M., Leadbeater W., Scheven B. A. (2013). Intravitreally transplanted dental pulp stem cells promote neuroprotection and axon regeneration of retinal ganglion cells after optic nerve injury. *Investigative Ophthalmology & Visual Science*.

[120] Holan V., Hermankova B., Krulova M., Zajicova A. (2019). Cytokine interplay among the diseased retina, inflammatory cells and mesenchymal stem cells—a clue to stem cell-based therapy. *World Journal of Stem Cells*.

[121] Johnson T. V., DeKorver N. W., Levasseur V. A. (2014). Identification of retinal ganglion cell neuroprotection conferred by platelet-derived growth factor through analysis of the mesenchymal stem cell secretome. *Brain*.

[122] Osborne A., Sanderson J., Martin K. R. (2018). Neuroprotective effects of human mesenchymal stem cells and platelet-derived growth factor on human retinal ganglion cells. *Stem Cells*.

[123] Holan V., Hermankova B., Bohacova P. (2016). Distinct immunoregulatory mechanisms in mesenchymal stem cells: role of the cytokine environment. *Stem Cell Reviews and Reports*.

[124] Kim J. Y., You Y. S., Kim S. H., Kwon O. W. (2017). Epiretinal membrane formation after intravitreal autologous stem cell implantation in a retinitis pigmentosa patient. *Retinal Cases and Brief Reports*.

[125] Kuriyan A. E., Albini T. A., Townsend J. H. (2017). Vision loss after intravitreal injection of autologous “stem cells” for AMD. *New England Journal of Medicine*.

[126] Dominici M., Le Blanc K., Mueller I. (2006). Minimal criteria for defining multipotent mesenchymal stromal cells. The International Society for Cellular Therapy position statement. *Cytotherapy*.

[127] Cuenca N., Fernandez-Sanchez L., Campello L. (2014). Cellular responses following retinal injuries and therapeutic approaches for neurodegenerative diseases. *Progress in Retinal and Eye Research*.

[128] Ratican S. E., Osborne A., Martin K. R. (2018). Progress in gene therapy to prevent retinal ganglion cell loss in glaucoma and Leber’s hereditary optic neuropathy. *Neural Plasticity*.

[129] Wilson A. M., Di Polo A. (2012). Gene therapy for retinal ganglion cell neuroprotection in glaucoma. *Gene Therapy*.

[130] Shimizu S., Lichter P. R., Johnson A. T. (2000). Age-dependent prevalence of mutations at the GLC1A locus in primary open-angle glaucoma. *American Journal of Ophthalmology*.

[131] Jain A., Zode G., Kasetti R. B. (2017). CRISPR-Cas9-based treatment of myocilin-associated glaucoma. *Proceedings of the National Academy of Sciences of the United States of America*.

[132] Sommerfeld M. T., Schweigreiter R., Barde Y. A., Hoppe E. (2000). Down-regulation of the neurotrophin receptor TrkB following ligand binding. Evidence for an involvement of the proteasome and differential regulation of TrkA and TrkB. *Journal of Biological Chemistry*.

[133] Osborne A., Khatib T. Z., Songra L. (2018). Neuroprotection of retinal ganglion cells by a novel gene therapy construct that achieves sustained enhancement of brain-derived neurotrophic factor/tropomyosin-related kinase receptor-B signaling. *Cell Death & Disease*.

[134] Lewis R. A., Christie W. C., Day D. G. (2017). Bimatoprost sustained-release implants for glaucoma therapy: 6-month results from a phase I/II clinical trial. *American Journal of Ophthalmology*.

[135] Brandt J. D., Sall K., DuBiner H. (2016). Six-month intraocular pressure reduction with a topical bimatoprost ocular insert: results of a phase II randomized controlled study. *Ophthalmology*.

[136] Brandt J. D., DuBiner H. B., Benza R. (2017). Long-term safety and efficacy of a sustained-release bimatoprost ocular ring. *Ophthalmology*.

[137] Xu J., Ge Y., Bu R. (2019). Co-delivery of latanoprost and timolol from micelles-laden contact lenses for the treatment of glaucoma. *Journal of Controlled Release*.

[138] Sleath B., Robin A. L., Covert D., Byrd J. E., Tudor G., Svarstad B. (2006). Patient-reported behavior and problems in using glaucoma medications. *Ophthalmology*.

[139] Kim C. Y., Park K. H., Ahn J. (2017). Treatment patterns and medication adherence of patients with glaucoma in South Korea. *The British Journal of Ophthalmology*.

[140] Okeke C. O., Quigley H. A., Jampel H. D. (2009). Adherence with topical glaucoma medication monitored electronically the Travatan dosing aid study. *Ophthalmology*.

[141] Tsai J. C., McClure C. A., Ramos S. E., Schlundt D. G., Pichert J. W. (2003). Compliance barriers in glaucoma: a systematic classification. *Journal of Glaucoma*.

[142] Olthoff C. M., Schouten J. S., van de Borne B. W., Webers C. A. (2005). Noncompliance with ocular hypotensive treatment in patients with glaucoma or ocular hypertension an evidence-based review. *Ophthalmology*.

[143] Tse A. P., Shah M., Jamal N., Shaikh A. (2016). Glaucoma treatment adherence at a United Kingdom general practice. *Eye*.

[144] Newman-Casey P. A., Niziol L. M., Gillespie B. W., Janz N. K., Lichter P. R., Musch D. C. (2020). The association between medication adherence and visual field progression in the collaborative initial glaucoma treatment study. *Ophthalmology*.

[145] Waterman H., Evans J. R., Gray T. A., Henson D., Harper R. (2013). Interventions for improving adherence to ocular hypotensive therapy. *Cochrane Database Systematic Reviews*.

[146] Juliana F. R., Kesse S., Boakye-Yiadom K. O., Veroniaina H., Wang H., Sun M. (2019). Promising approach in the treatment of glaucoma using nanotechnology and nanomedicine-based systems. *Molecules*.

[147] Occhiutto M. L., Maranhao R. C., Costa V. P., Konstas A. G. (2020). Nanotechnology for medical and surgical glaucoma therapy—a review. *Advances in Therapy*.

[148] McCarthy M. I., Abecasis G. R., Cardon L. R. (2008). Genome-wide association studies for complex traits: consensus, uncertainty and challenges. *Nature Reviews Genetics*.

[149] Khawaja A. P., Cooke Bailey J. N., Wareham N. J. (2018). Genome-wide analyses identify 68 new loci associated with intraocular pressure and improve risk prediction for primary open-angle glaucoma. *Nature Genetics*.

[150] Ramdas W. D., van Koolwijk L. M., Ikram M. K. (2010). A genome-wide association study of optic disc parameters. *PLoS Genetics*.

[151] Benson M. D., Khor C. C., Gage P. J., Lehmann O. J. (2017). A targeted approach to genome-wide studies reveals new genetic associations with central corneal thickness. *Molecular Vision*.

[152] Moroi S. E., Raoof D. A., Reed D. M., Zollner S., Qin Z., Richards J. E. (2009). Progress toward personalized medicine for glaucoma. *Expert Review of Ophthalmology*.

[153] Lopez C., Tucker S., Salameh T., Tucker C. (2018). An unsupervised machine learning method for discovering patient clusters based on genetic signatures. *Journal of Biomedical Informatics*.

[154] Moroi S. E., Reed D. M., Sanders D. S. (2019). Precision medicine to prevent glaucoma-related blindness. *Current Opinion in Ophthalmology*.

[155] Wu A., Khawaja A. P., Pasquale L. R., Stein J. D. (2019). A review of systemic medications that may modulate the risk of glaucoma. *Eye*.

[156] Yap T. E., Shamsher E., Guo L., Cordeiro M. F. (2019). Ophthalmic research lecture 2018: DARC as a potential surrogate marker. *Ophthalmic Research*.

[157] Agnifili L., Pieragostino D., Mastropasqua A. (2015). Molecular biomarkers in primary open-angle glaucoma: from noninvasive to invasive. *Progress in Brain Research*.

[158] Hindle A. G., Thoonen R., Jasien J. V. (2019). Identification of candidate miRNA biomarkers for glaucoma. *Investigative Ophthalmology & Visual Science*.

[159] Ong F. S., Kuo J. Z., Wu W. C. (2013). Personalized medicine in ophthalmology: from pharmacogenetic biomarkers to therapeutic and dosage optimization. *Journal of Personalized Medicine*.

[160] Huang A. S., Penteado R. C., Saha S. K. (2018). Fluorescein aqueous angiography in live normal human eyes. *Journal of Glaucoma*.

